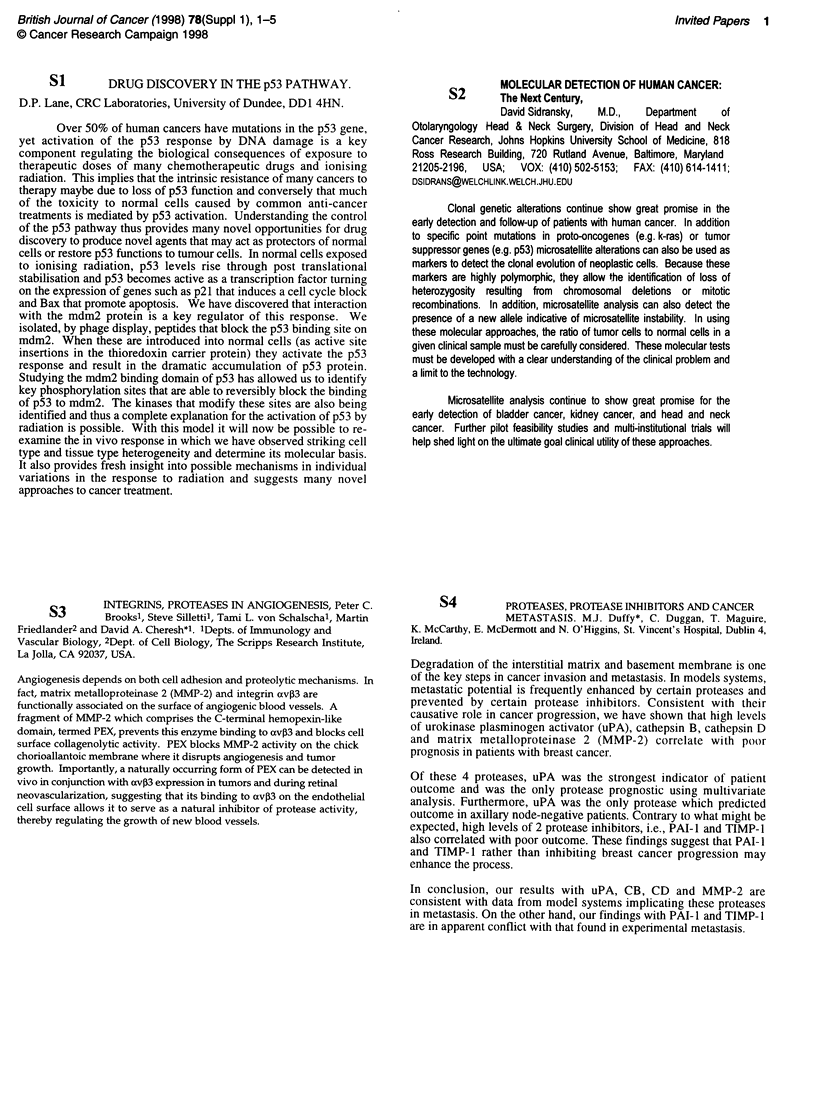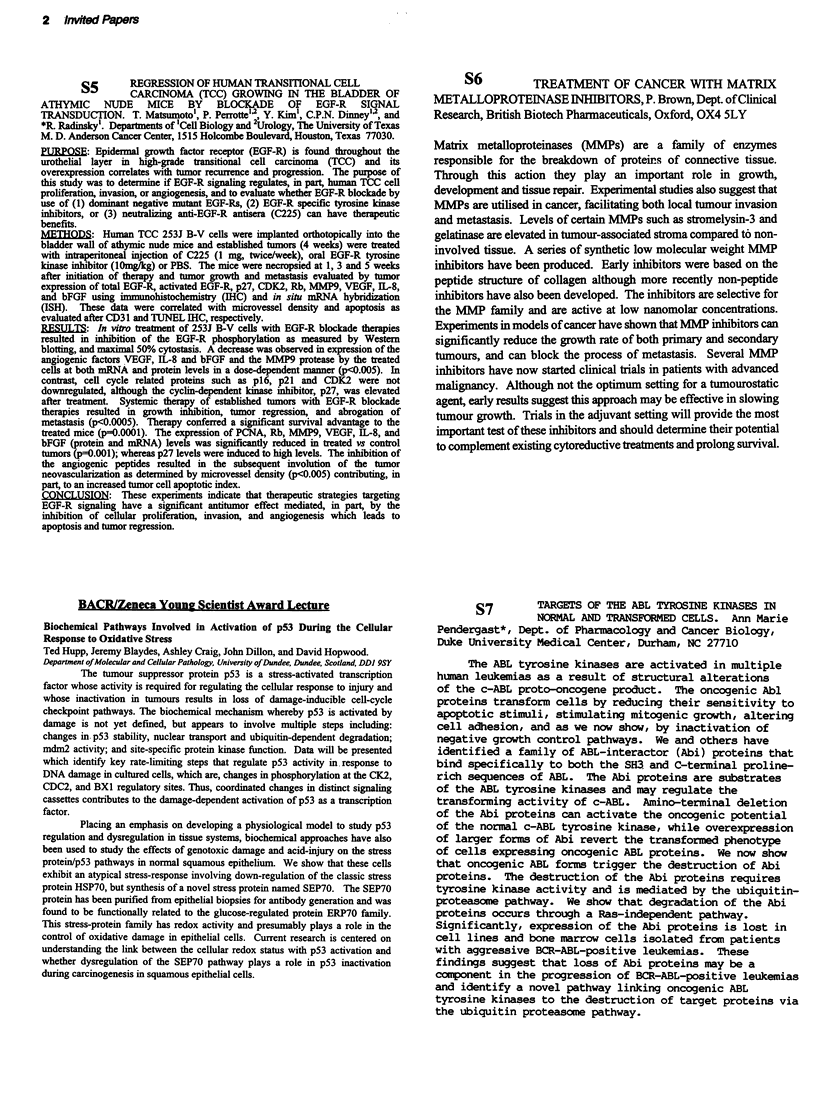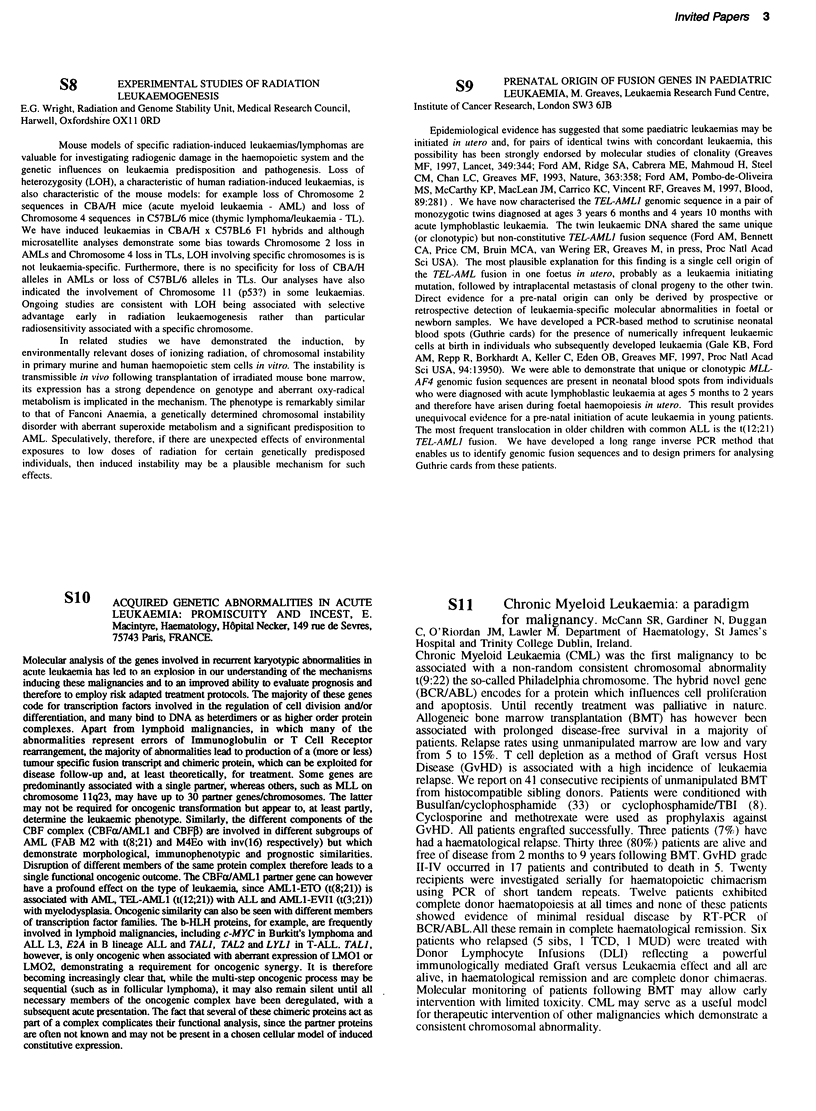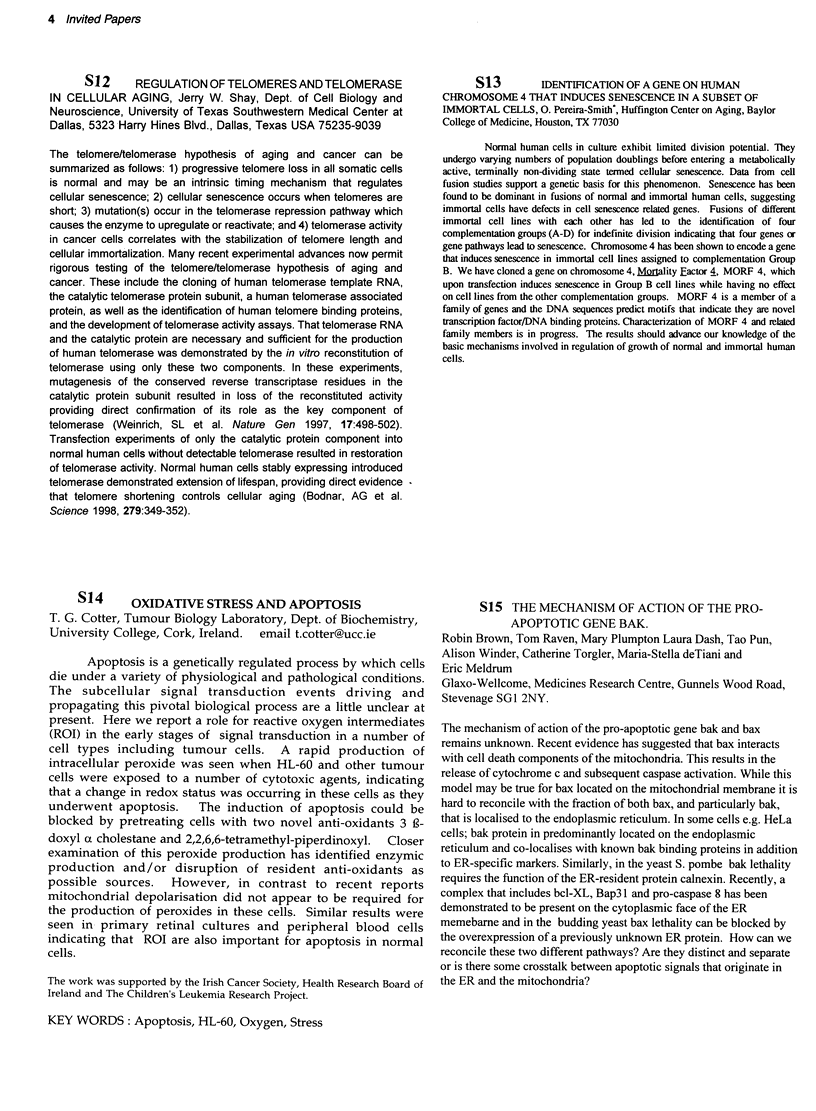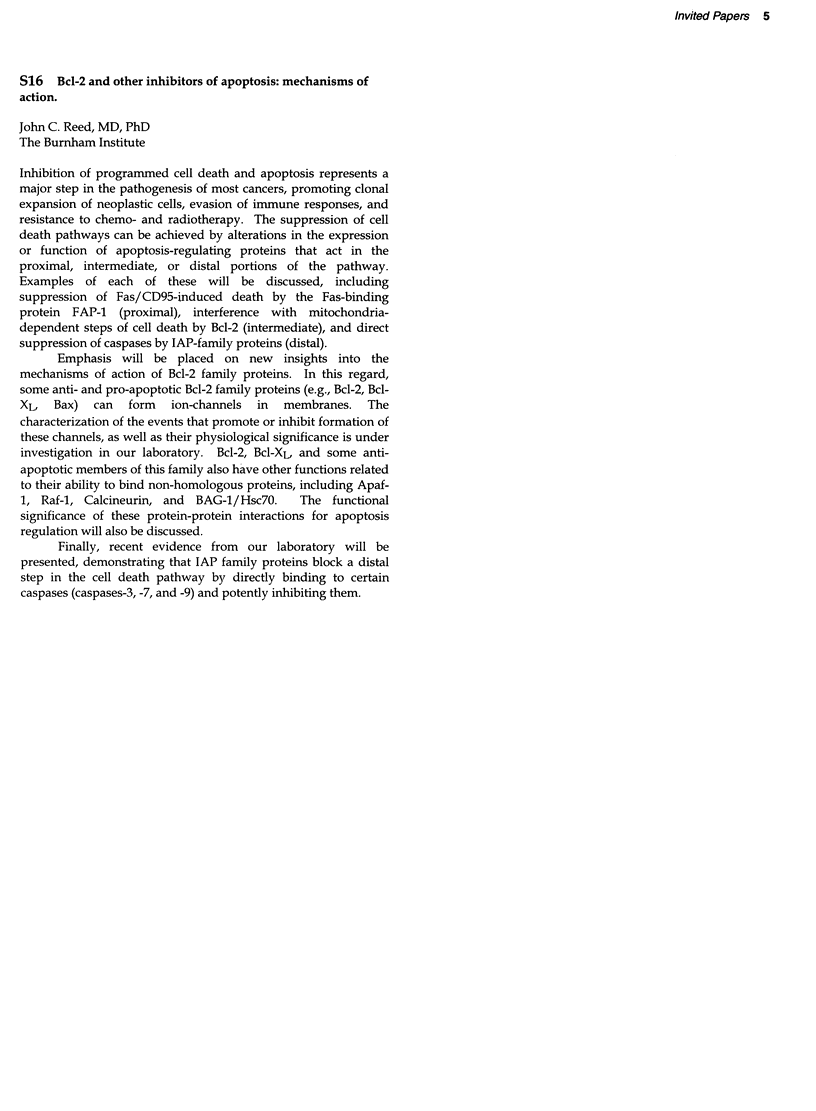# Joint British Association and Irish Association Cancer Research Meeting. Dublin, Ireland, 21-24 June 1998. Abstracts.

**Published:** 1998

**Authors:** 


© Cancer Research Campaign 1998

### S1 DRUG DISCOVERY IN THE p53 PATHWAY.

D.P. Lane, CRC Laboratories, University of Dundee, DD1 4HN.

Over 50% of human cancers have mutations in the p53 gene, yet activation of the p53 response by DNA damage is a key component regulating the biological consequences of exposure to therapeutic doses of many chemotherapeutic drugs and ionising radiation. This implies that the intrinsic resistance of many cancers to therapy maybe due to loss of p53 function and conversely that much of the toxicity to normal cells caused by common anti-cancer treatments is mediated by p53 activation. Understanding the control of the p53 pathway thus provides many novel opportunities for drug discovery to produce novel agents that may act as protectors of normal cells or restore p53 functions to tumour cells. In normal cells exposed to ionising radiation, p53 levels rise through post translational stabilisation and p53 becomes active as a transcription factor turning on the expression of genes such as p21 that induces a cell cycle block and Bax that promote apoptosis. We have discovered that interaction with the mdm2 protein is a key regulator of this response. We isolated, by phage display, peptides that block the p53 binding site on mdm2. When these are introduced into normal cells (as active site insertions in the thioredoxin carrier protein) they activate the p53 response and result in the dramatic accumulation of p53 protein. Studying the mdm2 binding domain of p53 has allowed us to identify key phosphorylation sites that are able to reversibly block the binding of p53 to mdm2. The kinases that modify these sites are also being identified and thus a complete explanation for the activation of p53 by radiation is possible. With this model it will now be possible to re-examine the in vivo response in which we have observed striking cell type and tissue type heterogeneity and determine its molecular basis. It also provides fresh insight into possible mechanisms in individual variations in the response to radiation and suggests many novel approaches to cancer treatment.

### S2 MOLECULAR DETECTION OF HUMAN CANCER: The Next Century,

David Sidransky, M.D., Department of Otolaryngology Head & Neck Surgery, Division of Head and Neck Cancer Research, Johns Hopkins University School of Medicine, 818 Ross Research Building, 720 Rutland Avenue, Baltimore, Maryland 21205-2196, USA; VOX: (410) 502-5153; FAX: (410) 614-1411; DSIDRANS@WELCHLINK.WELCH.JHU.EDU

Clonal genetic alterations continue show great promise in the early detection and follow-up of patients with human cancer. In addition to specific point mutations in proto-oncogenes (e.g. k-ras) or tumor suppressor genes (e.g. p53) microsatellite alterations can also be used as markers to detect the clonal evolution of neoplastic cells. Because these markers are highly polymorphic, they allow the identification of loss of heterozygosity resulting from chromosomal deletions or mitotic recombinations. In addition, microsatellite analysis can also detect the presence of a new allele indicative of microsatellite instability. In using these molecular approaches, the ratio of tumor cells to normal cells in a given clinical sample must be carefully considered. These molecular tests must be developed with a clear understanding of the clinical problem and a limit to the technology.

Microsatellite analysis continue to show great promise for the early detection of bladder cancer, kidney cancer, and head and neck cancer. Further pilot feasibility studies and multi-institutional trials will help shed light on the ultimate goal clinical utility of these approaches.

### S3 INTEGRINS, PROTEASES IN ANGIOGENESIS,

Peter C. Brooks[1], Steve Silletti[1], Tami L. von Schalscha[1], Martin Friedlander[2] and David A. Cheresh*[1]. [1]Depts. of Immunology and Vascular Biology, [2]Dept. of Cell Biology, The Scripps Research Institute, La Jolla, CA 92037, USA.

Angiogenesis depends on both cell adhesion and proteolytic mechanisms. In fact, matrix metalloproteinase 2 (MMP-2) and integrin αvβ3 are functionally associated on the surface of angiogenic blood vessels. A fragment of MMP-2 which comprises the C-terminal hemopexin-like domain, termed PEX, prevents this enzyme binding to αvβ3 and blocks cell surface collagenolytic activity. PEX blocks MMP-2 activity on the chick chorioallantoic membrane where it disrupts angiogenesis and tumor growth. Importantly, a naturally occurring form of PEX can be detected in vivo in conjunction with αvβ3 expression in tumors and during retinal neovascularization, suggesting that its binding to αvβ3 on the endothelial cell surface allows it to serve as a natural inhibitor of protease activity, thereby regulating the growth of new blood vessels.

### S4 PROTEASES, PROTEASE INHIBITORS AND CANCER METASTASIS.

M.J. Duffy*, C. Duggan, T. Maguire, K. McCarthy, E. McDermott and N. O'Higgins, St. Vincent's Hospital, Dublin 4, Ireland.

Degradation of the interstitial matrix and basement membrane is one of the key steps in cancer invasion and metastasis. In models systems, metastatic potential is frequently enhanced by certain proteases and prevented by certain protease inhibitors. Consistent with their causative role in cancer progression, we have shown that high levels of urokinase plasminogen activator (uPA), cathepsin B, cathepsin D and matrix metalloproteinase 2 (MMP-2) correlate with poor prognosis in patients with breast cancer.

Of these 4 proteases, uPA was the strongest indicator of patient outcome and was the only protease prognostic using multivariate analysis. Furthermore, uPA was the only protease which predicted outcome in axillary node-negative patients. Contrary to what might be expected, high levels of 2 protease inhibitors, i.e., PAI-1 and TIMP-1 also correlated with poor outcome. These findings suggest that PAI-1 and TIMP-1 rather than inhibiting breast cancer progression may enhance the process.

In conclusion, our results with uPA, CB, CD and MMP-2 are consistent with data from model systems implicating these proteases in metastasis. On the other hand, our findings with PAI-1 and TIMP-1 are in apparent conflict with that found in experimental metastasis.

### S5 REGRESSION OF HUMAN TRANSITIONAL CELL CARCINOMA (TCC) GROWING IN THE BLADDER OF ATHYMIC NUDE MICE BY BLOCKADE OF EGF-R SIGNAL TRANSDUCTION.

T. Matsumoto[1], P. Perrotte[1,2], Y. Kim[1], C.P.N. Dinney[1,2], and *R. Radinsky[1]. Departments of [1]Cell Biology and [2]Urology, The University of Texas M. D. Anderson Cancer Center, 1515 Holcombe Boulevard, Houston, Texas 77030.

PURPOSE: Epidermal growth factor receptor (EGF-R) is found throughout the urothelial layer in high-grade transitional cell carcinoma (TCC) and its overexpression correlates with tumor recurrence and progression. The purpose of this study was to determine if EGF-R signaling regulates, in part, human TCC cell proliferation, invasion, or angiogenesis, and to evaluate whether EGF-R blockade by use of (1) dominant negative mutant EGF-Rs, (2) EGF-R specific tyrosine kinase inhibitors, or (3) neutralizing anti-EGF-R antisera (C225) can have therapeutic benefits.
METHODS: Human TCC 253J B-V cells were implanted orthotopically into the bladder wall of athymic nude mice and established tumors (4 weeks) were treated with intraperitoneal injection of C225 (1 mg, twice/week), oral EGF-R tyrosine kinase inhibitor (10mg/kg) or PBS. The mice were necropsied at 1, 3 and 5 weeks after initiation of therapy and tumor growth and metastasis evaluated by tumor expression of total EGF-R, activated EGF-R, p27, CDK2, Rb, MMP9, VEGF, IL-8, and bFGF using immunohistochemistry (IHC) and *in situ* mRNA hybridization (ISH). These data were correlated with microvessel density and apoptosis as evaluated after CD31 and TUNEL IHC, respectively.
RESULTS: *In vitro* treatment of 253J B-V cells with EGF-R blockade therapies resulted in inhibition of the EGF-R phosphorylation as measured by Western blotting, and maximal 50% cytostasis. A decrease was observed in expression of the angiogenic factors VEGF, IL-8 and bFGF and the MMP9 protease by the treated cells at both mRNA and protein levels in a dose-dependent manner ($p<0.005$). In contrast, cell cycle related proteins such as p16, p21 and CDK2 were not downregulated, although the cyclin-dependent kinase inhibitor, p27, was elevated after treatment. Systemic therapy of established tumors with EGF-R blockade therapies resulted in growth inhibition, tumor regression, and abrogation of metastasis ($p<0.0005$). Therapy conferred a significant survival advantage to the treated mice ($p=0.0001$). The expression of PCNA, Rb, MMP9, VEGF, IL-8, and bFGF (protein and mRNA) levels was significantly reduced in treated *vs* control tumors ($p=0.001$); whereas p27 levels were induced to high levels. The inhibition of the angiogenic peptides resulted in the subsequent involution of the tumor neovascularization as determined by microvessel density ($p<0.005$) contributing, in part, to an increased tumor cell apoptotic index.
CONCLUSION: These experiments indicate that therapeutic strategies targeting EGF-R signaling have a significant antitumor effect mediated, in part, by the inhibition of cellular proliferation, invasion, and angiogenesis which leads to apoptosis and tumor regression.

### S6 TREATMENT OF CANCER WITH MATRIX METALLOPROTEINASE INHIBITORS,

P. Brown, Dept. of Clinical Research, British Biotech Pharmaceuticals, Oxford, OX4 5LY

Matrix metalloproteinases (MMPs) are a family of enzymes responsible for the breakdown of proteins of connective tissue. Through this action they play an important role in growth, development and tissue repair. Experimental studies also suggest that MMPs are utilised in cancer, facilitating both local tumour invasion and metastasis. Levels of certain MMPs such as stromelysin-3 and gelatinase are elevated in tumour-associated stroma compared to non-involved tissue. A series of synthetic low molecular weight MMP inhibitors have been produced. Early inhibitors were based on the peptide structure of collagen although more recently non-peptide inhibitors have also been developed. The inhibitors are selective for the MMP family and are active at low nanomolar concentrations. Experiments in models of cancer have shown that MMP inhibitors can significantly reduce the growth rate of both primary and secondary tumours, and can block the process of metastasis. Several MMP inhibitors have now started clinical trials in patients with advanced malignancy. Although not the optimum setting for a tumourostatic agent, early results suggest this approach may be effective in slowing tumour growth. Trials in the adjuvant setting will provide the most important test of these inhibitors and should determine their potential to complement existing cytoreductive treatments and prolong survival.

### BACR/Zeneca Young Scientist Award Lecture

### Biochemical Pathways Involved in Activation of p53 During the Cellular Response to Oxidative Stress

Ted Hupp, Jeremy Blaydes, Ashley Craig, John Dillon, and David Hopwood.
*Department of Molecular and Cellular Pathology, University of Dundee, Dundee, Scotland, DD1 9SY*

The tumour suppressor protein p53 is a stress-activated transcription factor whose activity is required for regulating the cellular response to injury and whose inactivation in tumours results in loss of damage-inducible cell-cycle checkpoint pathways. The biochemical mechanism whereby p53 is activated by damage is not yet defined, but appears to involve multiple steps including: changes in p53 stability, nuclear transport and ubiquitin-dependent degradation; mdm2 activity; and site-specific protein kinase function. Data will be presented which identify key rate-limiting steps that regulate p53 activity in response to DNA damage in cultured cells, which are, changes in phosphorylation at the CK2, CDC2, and BX1 regulatory sites. Thus, coordinated changes in distinct signaling cassettes contributes to the damage-dependent activation of p53 as a transcription factor.

Placing an emphasis on developing a physiological model to study p53 regulation and dysregulation in tissue systems, biochemical approaches have also been used to study the effects of genotoxic damage and acid-injury on the stress protein/p53 pathways in normal squamous epithelium. We show that these cells exhibit an atypical stress-response involving down-regulation of the classic stress protein HSP70, but synthesis of a novel stress protein named SEP70. The SEP70 protein has been purified from epithelial biopsies for antibody generation and was found to be functionally related to the glucose-regulated protein ERP70 family. This stress-protein family has redox activity and presumably plays a role in the control of oxidative damage in epithelial cells. Current research is centered on understanding the link between the cellular redox status with p53 activation and whether dysregulation of the SEP70 pathway plays a role in p53 inactivation during carcinogenesis in squamous epithelial cells.

### S7 TARGETS OF THE ABL TYROSINE KINASES IN NORMAL AND TRANSFORMED CELLS.

Ann Marie Pendergast*, Dept. of Pharmacology and Cancer Biology, Duke University Medical Center, Durham, NC 27710

The ABL tyrosine kinases are activated in multiple human leukemias as a result of structural alterations of the c-ABL proto-oncogene product. The oncogenic Abl proteins transform cells by reducing their sensitivity to apoptotic stimuli, stimulating mitogenic growth, altering cell adhesion, and as we now show, by inactivation of negative growth control pathways. We and others have identified a family of ABL-interactor (Abi) proteins that bind specifically to both the SH3 and C-terminal proline-rich sequences of ABL. The Abi proteins are substrates of the ABL tyrosine kinases and may regulate the transforming activity of c-ABL. Amino-terminal deletion of the Abi proteins can activate the oncogenic potential of the normal c-ABL tyrosine kinase, while overexpression of larger forms of Abi revert the transformed phenotype of cells expressing oncogenic ABL proteins. We now show that oncogenic ABL forms trigger the destruction of Abi proteins. The destruction of the Abi proteins requires tyrosine kinase activity and is mediated by the ubiquitin-proteasome pathway. We show that degradation of the Abi proteins occurs through a Ras-independent pathway. Significantly, expression of the Abi proteins is lost in cell lines and bone marrow cells isolated from patients with aggressive BCR-ABL-positive leukemias. These findings suggest that loss of Abi proteins may be a component in the progression of BCR-ABL-positive leukemias and identify a novel pathway linking oncogenic ABL tyrosine kinases to the destruction of target proteins via the ubiquitin proteasome pathway.

### S8 EXPERIMENTAL STUDIES OF RADIATION LEUKAEMOGENESIS

E.G. Wright, Radiation and Genome Stability Unit, Medical Research Council, Harwell, Oxfordshire OX11 0RD

Mouse models of specific radiation-induced leukaemias/lymphomas are valuable for investigating radiogenic damage in the haemopoietic system and the genetic influences on leukaemia predisposition and pathogenesis. Loss of heterozygosity (LOH), a characteristic of human radiation-induced leukaemias, is also characteristic of the mouse models: for example loss of Chromosome 2 sequences in CBA/H mice (acute myeloid leukaemia - AML) and loss of Chromosome 4 sequences in C57BL/6 mice (thymic lymphoma/leukaemia - TL). We have induced leukaemias in CBA/H x C57BL6 F1 hybrids and although microsatellite analyses demonstrate some bias towards Chromosome 2 loss in AMLs and Chromosome 4 loss in TLs, LOH involving specific chromosomes is is not leukaemia-specific. Furthermore, there is no specificity for loss of CBA/H alleles in AMLs or loss of C57BL/6 alleles in TLs. Our analyses have also indicated the involvement of Chromosome 11 (p53?) in some leukaemias. Ongoing studies are consistent with LOH being associated with selective advantage early in radiation leukaemogenesis rather than particular radiosensitivity associated with a specific chromosome.

In related studies we have demonstrated the induction, by environmentally relevant doses of ionizing radiation, of chromosomal instability in primary murine and human haemopoietic stem cells *in vitro*. The instability is transmissible *in vivo* following transplantation of irradiated mouse bone marrow, its expression has a strong dependence on genotype and aberrant oxy-radical metabolism is implicated in the mechanism. The phenotype is remarkably similar to that of Fanconi Anaemia, a genetically determined chromosomal instability disorder with aberrant superoxide metabolism and a significant predisposition to AML. Speculatively, therefore, if there are unexpected effects of environmental exposures to low doses of radiation for certain genetically predisposed individuals, then induced instability may be a plausible mechanism for such effects.

### S9 PRENATAL ORIGIN OF FUSION GENES IN PAEDIATRIC LEUKAEMIA, M. Greaves, Leukaemia Research Fund Centre, Institute of Cancer Research, London SW3 6JB

Epidemiological evidence has suggested that some paediatric leukaemias may be initiated *in utero* and, for pairs of identical twins with concordant leukaemia, this possibility has been strongly endorsed by molecular studies of clonality (Greaves MF, 1997, Lancet, 349:344; Ford AM, Ridge SA, Cabrera ME, Mahmoud H, Steel CM, Chan LC, Greaves MF, 1993, Nature, 363:358; Ford AM, Pombo-de-Oliveira MS, McCarthy KP, MacLean JM, Carrico KC, Vincent RF, Greaves M, 1997, Blood, 89:281) . We have now characterised the *TEL-AML1* genomic sequence in a pair of monozygotic twins diagnosed at ages 3 years 6 months and 4 years 10 months with acute lymphoblastic leukaemia. The twin leukaemic DNA shared the same unique (or clonotypic) but non-constitutive *TEL-AML1* fusion sequence (Ford AM, Bennett CA, Price CM, Bruin MCA, van Wering ER, Greaves M, in press, Proc Natl Acad Sci USA). The most plausible explanation for this finding is a single cell origin of the *TEL-AML* fusion in one foetus *in utero*, probably as a leukaemia initiating mutation, followed by intraplacental metastasis of clonal progeny to the other twin. Direct evidence for a pre-natal origin can only be derived by prospective or retrospective detection of leukaemia-specific molecular abnormalities in foetal or newborn samples. We have developed a PCR-based method to scrutinise neonatal blood spots (Guthrie cards) for the presence of numerically infrequent leukaemic cells at birth in individuals who subsequently developed leukaemia (Gale KB, Ford AM, Repp R, Borkhardt A, Keller C, Eden OB, Greaves MF, 1997, Proc Natl Acad Sci USA, 94:13950). We were able to demonstrate that unique or clonotypic *MLL-AF4* genomic fusion sequences are present in neonatal blood spots from individuals who were diagnosed with acute lymphoblastic leukaemia at ages 5 months to 2 years and therefore have arisen during foetal haemopoiesis *in utero*. This result provides unequivocal evidence for a pre-natal initiation of acute leukaemia in young patients. The most frequent translocation in older children with common ALL is the t(12;21) *TEL-AML1* fusion. We have developed a long range inverse PCR method that enables us to identify genomic fusion sequences and to design primers for analysing Guthrie cards from these patients.

### S10 ACQUIRED GENETIC ABNORMALITIES IN ACUTE LEUKAEMIA: PROMISCUITY AND INCEST, E. Macintyre, Haematology, Hôpital Necker, 149 rue de Sevres, 75743 Paris, FRANCE.

Molecular analysis of the genes involved in recurrent karyotypic abnormalities in acute leukaemia has led to an explosion in our understanding of the mechanisms inducing these malignancies and to an improved ability to evaluate prognosis and therefore to employ risk adapted treatment protocols. The majority of these genes code for transcription factors involved in the regulation of cell division and/or differentiation, and many bind to DNA as heterdimers or as higher order protein complexes. Apart from lymphoid malignancies, in which many of the abnormalities represent errors of Immunoglobulin or T Cell Receptor rearrangement, the majority of abnormalities lead to production of a (more or less) tumour specific fusion transcript and chimeric protein, which can be exploited for disease follow-up and, at least theoretically, for treatment. Some genes are predominantly associated with a single partner, whereas others, such as MLL on chromosome 11q23, may have up to 30 partner genes/chromosomes. The latter may not be required for oncogenic transformation but appear to, at least partly, determine the leukaemic phenotype. Similarly, the different components of the CBF complex (CBFα/AML1 and CBFβ) are involved in different subgroups of AML (FAB M2 with t(8;21) and M4Eo with inv(16) respectively) but which demonstrate morphological, immunophenotypic and prognostic similarities. Disruption of different members of the same protein complex therefore leads to a single functional oncogenic outcome. The CBFα/AML1 partner gene can however have a profound effect on the type of leukaemia, since AML1-ETO (t(8;21)) is associated with AML, TEL-AML1 (t(12;21)) with ALL and AML1-EVI1 (t(3;21)) with myelodysplasia. Oncogenic similarity can also be seen with different members of transcription factor families. The b-HLH proteins, for example, are frequently involved in lymphoid malignancies, including *c-MYC* in Burkitt's lymphoma and ALL L3, *E2A* in B lineage ALL and *TAL1*, *TAL2* and *LYL1* in T-ALL. *TAL1*, however, is only oncogenic when associated with aberrant expression of LMO1 or LMO2, demonstrating a requirement for oncogenic synergy. It is therefore becoming increasingly clear that, while the multi-step oncogenic process may be sequential (such as in follicular lymphoma), it may also remain silent until all necessary members of the oncogenic complex have been deregulated, with a subsequent acute presentation. The fact that several of these chimeric proteins act as part of a complex complicates their functional analysis, since the partner proteins are often not known and may not be present in a chosen cellular model of induced constitutive expression.

### S11 Chronic Myeloid Leukaemia: a paradigm for malignancy. McCann SR, Gardiner N, Duggan C, O'Riordan JM, Lawler M. Department of Haematology, St James's Hospital and Trinity College Dublin, Ireland.

Chronic Myeloid Leukaemia (CML) was the first malignancy to be associated with a non-random consistent chromosomal abnormality t(9:22) the so-called Philadelphia chromosome. The hybrid novel gene (BCR/ABL) encodes for a protein which influences cell proliferation and apoptosis. Until recently treatment was palliative in nature. Allogeneic bone marrow transplantation (BMT) has however been associated with prolonged disease-free survival in a majority of patients. Relapse rates using unmanipulated marrow are low and vary from 5 to 15%. T cell depletion as a method of Graft versus Host Disease (GvHD) is associated with a high incidence of leukaemia relapse. We report on 41 consecutive recipients of unmanipulated BMT from histocompatible sibling donors. Patients were conditioned with Busulfan/cyclophosphamide (33) or cyclophosphamide/TBI (8). Cyclosporine and methotrexate were used as prophylaxis against GvHD. All patients engrafted successfully. Three patients (7%) have had a haematological relapse. Thirty three (80%) patients are alive and free of disease from 2 months to 9 years following BMT. GvHD grade II-IV occurred in 17 patients and contributed to death in 5. Twenty recipients were investigated serially for haematopoietic chimaerism using PCR of short tandem repeats. Twelve patients exhibited complete donor haematopoiesis at all times and none of these patients showed evidence of minimal residual disease by RT-PCR of BCR/ABL.All these remain in complete haematological remission. Six patients who relapsed (5 sibs, 1 TCD, 1 MUD) were treated with Donor Lymphocyte Infusions (DLI) reflecting a powerful immunologically mediated Graft versus Leukaemia effect and all are alive, in haematological remission and are complete donor chimaeras. Molecular monitoring of patients following BMT may allow early intervention with limited toxicity. CML may serve as a useful model for therapeutic intervention of other malignancies which demonstrate a consistent chromosomal abnormality.

### S12 REGULATION OF TELOMERES AND TELOMERASE IN CELLULAR AGING

Jerry W. Shay, Dept. of Cell Biology and Neuroscience, University of Texas Southwestern Medical Center at Dallas, 5323 Harry Hines Blvd., Dallas, Texas USA 75235-9039

The telomere/telomerase hypothesis of aging and cancer can be summarized as follows: 1) progressive telomere loss in all somatic cells is normal and may be an intrinsic timing mechanism that regulates cellular senescence; 2) cellular senescence occurs when telomeres are short; 3) mutation(s) occur in the telomerase repression pathway which causes the enzyme to upregulate or reactivate; and 4) telomerase activity in cancer cells correlates with the stabilization of telomere length and cellular immortalization. Many recent experimental advances now permit rigorous testing of the telomere/telomerase hypothesis of aging and cancer. These include the cloning of human telomerase template RNA, the catalytic telomerase protein subunit, a human telomerase associated protein, as well as the identification of human telomere binding proteins, and the development of telomerase activity assays. That telomerase RNA and the catalytic protein are necessary and sufficient for the production of human telomerase was demonstrated by the *in vitro* reconstitution of telomerase using only these two components. In these experiments, mutagenesis of the conserved reverse transcriptase residues in the catalytic protein subunit resulted in loss of the reconstituted activity providing direct confirmation of its role as the key component of telomerase (Weinrich, SL et al. *Nature Gen* 1997, **17**:498-502). Transfection experiments of only the catalytic protein component into normal human cells without detectable telomerase resulted in restoration of telomerase activity. Normal human cells stably expressing introduced telomerase demonstrated extension of lifespan, providing direct evidence that telomere shortening controls cellular aging (Bodnar, AG et al. *Science* 1998, **279**:349-352).

### S13 IDENTIFICATION OF A GENE ON HUMAN CHROMOSOME 4 THAT INDUCES SENESCENCE IN A SUBSET OF IMMORTAL CELLS

O. Pereira-Smith*, Huffington Center on Aging, Baylor College of Medicine, Houston, TX 77030

Normal human cells in culture exhibit limited division potential. They undergo varying numbers of population doublings before entering a metabolically active, terminally non-dividing state termed cellular senescence. Data from cell fusion studies support a genetic basis for this phenomenon. Senescence has been found to be dominant in fusions of normal and immortal human cells, suggesting immortal cells have defects in cell senescence related genes. Fusions of different immortal cell lines with each other has led to the identification of four complementation groups (A-D) for indefinite division indicating that four genes or gene pathways lead to senescence. Chromosome 4 has been shown to encode a gene that induces senescence in immortal cell lines assigned to complementation Group B. We have cloned a gene on chromosome 4, Mortality Factor 4, MORF 4, which upon transfection induces senescence in Group B cell lines while having no effect on cell lines from the other complementation groups. MORF 4 is a member of a family of genes and the DNA sequences predict motifs that indicate they are novel transcription factor/DNA binding proteins. Characterization of MORF 4 and related family members is in progress. The results should advance our knowledge of the basic mechanisms involved in regulation of growth of normal and immortal human cells.

### S14 OXIDATIVE STRESS AND APOPTOSIS

T. G. Cotter, Tumour Biology Laboratory, Dept. of Biochemistry, University College, Cork, Ireland. email t.cotter@ucc.ie

Apoptosis is a genetically regulated process by which cells die under a variety of physiological and pathological conditions. The subcellular signal transduction events driving and propagating this pivotal biological process are a little unclear at present. Here we report a role for reactive oxygen intermediates (ROI) in the early stages of signal transduction in a number of cell types including tumour cells. A rapid production of intracellular peroxide was seen when HL-60 and other tumour cells were exposed to a number of cytotoxic agents, indicating that a change in redox status was occurring in these cells as they underwent apoptosis. The induction of apoptosis could be blocked by pretreating cells with two novel anti-oxidants 3 ß-doxyl α cholestane and 2,2,6,6-tetramethyl-piperdinoxyl. Closer examination of this peroxide production has identified enzymic production and/or disruption of resident anti-oxidants as possible sources. However, in contrast to recent reports mitochondrial depolarisation did not appear to be required for the production of peroxides in these cells. Similar results were seen in primary retinal cultures and peripheral blood cells indicating that ROI are also important for apoptosis in normal cells.

The work was supported by the Irish Cancer Society, Health Research Board of Ireland and The Children's Leukemia Research Project.

KEY WORDS : Apoptosis, HL-60, Oxygen, Stress

### S15 THE MECHANISM OF ACTION OF THE PRO-APOPTOTIC GENE BAK.

Robin Brown, Tom Raven, Mary Plumpton Laura Dash, Tao Pun, Alison Winder, Catherine Torgler, Maria-Stella deTiani and Eric Meldrum
Glaxo-Wellcome, Medicines Research Centre, Gunnels Wood Road, Stevenage SG1 2NY.

The mechanism of action of the pro-apoptotic gene bak and bax remains unknown. Recent evidence has suggested that bax interacts with cell death components of the mitochondria. This results in the release of cytochrome c and subsequent caspase activation. While this model may be true for bax located on the mitochondrial membrane it is hard to reconcile with the fraction of both bax, and particularly bak, that is localised to the endoplasmic reticulum. In some cells e.g. HeLa cells; bak protein in predominantly located on the endoplasmic reticulum and co-localises with known bak binding proteins in addition to ER-specific markers. Similarly, in the yeast S. pombe bak lethality requires the function of the ER-resident protein calnexin. Recently, a complex that includes bcl-XL, Bap31 and pro-caspase 8 has been demonstrated to be present on the cytoplasmic face of the ER memebarne and in the budding yeast bax lethality can be blocked by the overexpression of a previously unknown ER protein. How can we reconcile these two different pathways? Are they distinct and separate or is there some crosstalk between apoptotic signals that originate in the ER and the mitochondria?

### S16 Bcl-2 and other inhibitors of apoptosis: mechanisms of action.

John C. Reed, MD, PhD
The Burnham Institute

Inhibition of programmed cell death and apoptosis represents a major step in the pathogenesis of most cancers, promoting clonal expansion of neoplastic cells, evasion of immune responses, and resistance to chemo- and radiotherapy. The suppression of cell death pathways can be achieved by alterations in the expression or function of apoptosis-regulating proteins that act in the proximal, intermediate, or distal portions of the pathway. Examples of each of these will be discussed, including suppression of Fas/CD95-induced death by the Fas-binding protein FAP-1 (proximal), interference with mitochondria-dependent steps of cell death by Bcl-2 (intermediate), and direct suppression of caspases by IAP-family proteins (distal).

Emphasis will be placed on new insights into the mechanisms of action of Bcl-2 family proteins. In this regard, some anti- and pro-apoptotic Bcl-2 family proteins (e.g., Bcl-2, Bcl-$X_L$, Bax) can form ion-channels in membranes. The characterization of the events that promote or inhibit formation of these channels, as well as their physiological significance is under investigation in our laboratory. Bcl-2, Bcl-$X_L$, and some anti-apoptotic members of this family also have other functions related to their ability to bind non-homologous proteins, including Apaf-1, Raf-1, Calcineurin, and BAG-1/Hsc70. The functional significance of these protein-protein interactions for apoptosis regulation will also be discussed.

Finally, recent evidence from our laboratory will be presented, demonstrating that IAP family proteins block a distal step in the cell death pathway by directly binding to certain caspases (caspases-3, -7, and -9) and potently inhibiting them.